# Natural killer cell status and tolerance in mouse and human bacterial sepsis

**DOI:** 10.1186/cc11771

**Published:** 2012-11-14

**Authors:** F Souza-Fonseca-Guimaraes, M Parlato, F Philippart, B Misset, JM Cavaillon, M Adib-Conquy

**Affiliations:** 1 Institut Pasteur, Paris, France; 2 Groupe hospitalier Paris Saint Joseph, Paris, France

## Background

As sensors of infection, innate immune cells are able to recognize pathogen-associated molecular patterns by receptors such as Toll-like receptors (TLRs). Natural killer (NK) cells contribute to inflammatory processes by producing proinflammatory cytokines such as IFNγ and GM-CSF [1]. Our aim was to characterize the immune status of NK cells in a murine model of sepsis and in patients with systemic inflammatory response syndrome (SIRS) and sepsis.

## Methods

Cecal puncture (CP) was employed as a murine model of polymicrobial sepsis. TLR expression in murine and human NK cells was studied by flow cytometry. *Ex vivo *IFNγ production was analyzed either by ELISA or by flow cytometry.

## Results

In mice, the expression of TLR2 and TLR4 in spleen NK cells is mainly intracellular, similarly to TLR9. *In vitro *cell responsiveness of purified NK cells to TLR2, TLR4 or TLR9 agonists, in synergy with accessory cytokines (IL-2, IL-15 and IL-18), allowed a significant production of IFNγ and GM-CSF. In contrast, NK cells, purified from spleen of mice with sepsis, showed a dramatic reduction of their capacity to produce cytokines in response to TLR agonists. Depletion of regulatory T cells (Tregs) before CP led to a complete reversion of NK cell tolerance to TLR agonists. IL-10 and TGF-β1 are two main inhibitory cytokines produced by Tregs. We showed *in vivo*, using IL-10 knockout mice and by inhibiting TGF-βR signaling, that the tolerization mechanism of NK cells was mostly mediated by TGF-β [2]. In humans, the expression of TLR2, TLR4 and TLR9 in peripheral blood NK cells (both CD3^-^CD56^high ^and CD3^-^CD56^dim ^subsets) was mainly intracellular. The *ex vivo *responsiveness of the blood NK cells to their agonists in synergy with accessory cytokines (IL-15 and IL-18), allowed a significant secretion of IFNγ. Similar to the murine model of sepsis, in SIRS and sepsis patients the secretion of IFNγ by NK cells was significantly decreased.

## Conclusion

NK cells express TLR2 and TLR4 intracellularly, as already reported for other cell types (epithelial, endothelial, and dendritic cells). Furthermore, NK cells undergo tolerance to TLR agonists during SIRS or sepsis, as already described for monocytes in these clinical settings.
